# Supplementary material for: Mental Health and Wellbeing of 9–12-year-old Children in Northern Canada Before the COVID-19 Pandemic and After the First Lockdown
Source: Int J Public Health. 2021 Sep 1;66:1604219. doi: 10.3389/ijph.2021.1604219 (PMC8441596; doi:10.3389/ijph.2021.1604219)
Supplement: Supplementary file 2 [file Table1.docx]

**Supplementary materials**

**Table S1.** Factor loadings for 12 mental health and wellbeing questions completed by 9-12 year old school students, separately for girls and boys, Canada, 2018-2020

|  | **Girls** | | | **Boys** | | |
| --- | --- | --- | --- | --- | --- | --- |
|  | **Factor 1** | **Factor 2** | **Factor 3** | **Factor 1** | **Factor 2** | **Factor 3** |
| **Negatively stated items** |  |  | .. |  |  |  |
| I feel unhappy or sad |  |  | 0.64 |  |  | 0.59 |
| I worry a lot |  |  | 0.57 |  |  | 0.55 |
| I am in trouble with my teacher(s) |  |  | 0.34 |  | 0.66 |  |
| I have trouble paying attention |  |  | 0.56 |  | 0.46 |  |
| I have trouble enjoying myself |  |  | 0.39 |  | 0.31 |  |
| **Positively stated items** |  |  |  |  |  |  |
| My future looks good to me |  | 0.69 |  | 0.59 |  |  |
| I like the way I look | 0.75 |  |  | 0.68 |  |  |
| I like myself | 0.72 |  |  | 0.69 |  |  |
| I feel like I belong at school |  | 0.38 |  | 0.57 |  |  |
| I do well in my schoolwork |  | 0.39 |  | 0.43 |  |  |
| I feel like I have many friends |  | 0.43 |  | 0.45 |  |  |
| I have someone I trust to go to for advice |  | 0.42 |  | 0.57 |  |  |
